# Increased effective mass and carrier concentration responsible for the improved thermoelectric performance of the nominal compound Cu_2_Ga_4_Te_7_ with Sb substitution for Cu†

**DOI:** 10.1039/c8ra03704c

**Published:** 2018-06-14

**Authors:** Jiaolin Cui, Gemei Cai, Wei Ren

**Affiliations:** School of Materials & Chemical Engineering, Ningbo University of Technology Ningbo 315016 China cuijiaolin@163.com; School of Materials Science and Engineering, Central South University Changsha 410083 China caigemei@csu.edu.cn

## Abstract

Although the ternary chalcopyrite compound Cu_2_Ga_4_Te_7_ has relatively high thermal conductivity and electrical resistivity, it has a high carrier concentration, thus making it a good thermoelectric candidate. In this work we substitute Sb for Cu in this compound, aiming at engineering both the electrical and thermal properties. Rietveld refinement revealed that the nominal compounds Cu_2−*x*_Sb_*x*_Ga_4_Te_7_ (*x* = 0–0.1) crystallize with the crystal structure of CuGaTe_2_ with the real compositions deviating from those of their nominal ones. Besides, Sb resides in Cu sites, which increases both the effective mass and the Hall carrier concentration. Therefore, the Seebeck coefficient increases at high temperatures, and the lattice thermal conductivity reduces due to increased phonon scattering from point defects and electron–phonon interactions. As a consequence, the thermoelectric (TE) performance improves with the highest TE figure of merit (*ZT*) of 0.58 at 803 K. This value is about 0.21 higher than that of the pristine Cu_2_Ga_4_Te_7_.

## Abstract 2

A replacement of Sb for Cu in the nominal compound Cu_2_Ga_4_Te_7_ increases the effective carrier mass and the scattering of phonons.

## Graphical abstract

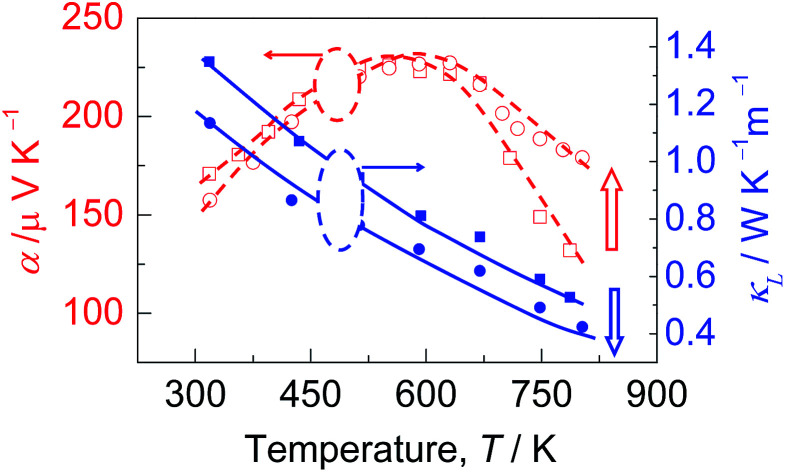

## 1. Introduction

Thermoelectric (TE) materials can directly convert heat into electricity and *vice versa*. The efficiency of TE devices is strongly dependent on the performance of materials, *i.e.* the dimensionless figure of merit (*ZT*), which is defined by the relation, *ZT* = *Tα*^2^*σ*/*κ*. Here the parameters *T*, *α*, *σ* and *κ* are the absolute temperature, the Seebeck coefficient, and the electrical and total thermal conductivity respectively. In order to enhance the *ZT* value, one should increase the power factor PF, PF = *α*^2^*σ*, and reduce the *κ* value that is mainly contributed by the lattice (*κ*_L_) and electronic (*κ*_e_) components. Since the three physical parameters *α*, *σ* and *κ*_e_ are closely related to the carrier concentration, it is not easy to control them separately. The strategies to enhance the *ZT* value proposed in recent years are those like nanostructure^1–3^ and band structure engineering,^4–6^ liquid-like thermoelectric explorations,^7,8^ as well as the study of magnetoelectric interactions,^9,10^*etc.* These approaches either improve the power factor (*α*^2^*σ*) or reduce the lattice component (*κ*_L_), provided that the carrier concentration is optimized.^11^ In addition to the above approaches, there is a strong need to develop new TE materials.

Ternary I–III–VI compounds have been paid much attention in recent years for thermoelectric applications.^12–14^ Owing to their inherent crystal or band structures,^15–17^ one often employs approaches, such as doping or solid solution formation, to improve their TE performances.^18–20^ The typical doping elements are those such as Ag,^14,20^ Sb,^21,22^ Mn^19^*etc.*, since impurity doping effectively engineers the band structures and introduces lattice disorder, thus increasing the carrier concentration and phonon scattering.

Cu_2_Ga_4_Te_7_ is one of the I–III–VI ternary compounds with two crystal structures. One is cubic (zinc blende) and the other is a tetragonal (chalcopyrite) structure.^23^ Because of the one-seventh cation vacancies in its unit cell, this compound is usually a p-type semiconductor with a Hall carrier concentration (*n*_H_) of 1.0 × 10^18^ to 8.3 × 10^19^ cm^−3^.^24,25^ Although the *n*_H_ value is close to the optimal one with respect to the TE performance,^26^ the compound Cu_2_Ga_4_Te_7_ has a relatively high electrical resistivity and thermal conductivity.^24,25^ It was reported that the highest *ZT* value of Cu_2_Ga_4_Te_7_ is less than 0.47 at ∼770 K,^25,27^ and 0.64 at 940 K.^28^ Therefore, there is a requirement to further improve its TE performance.

Inspired by an effective hybridization of active Sb-5p orbital with those of Cu-4s and Te-5p in the valence band in the newly developed Cu-deficient Cu_18_Ga_25_Te_50_ (cation/anion = 0.86),^21^ which unpins the Fermi level and enhances the carrier concentration,^21^ we postulate that an incorporation of Sb in Cu_2_Ga_4_Te_7_ with an almost identical cation/anion ratio (0.857) might also have a profound impact on the structure and transport properties. However, unlike a proper replacement of Sb for Te in Cu_18_Ga_25_Te_50_,^21^ in this work we design the chemical compositions with a replacement of Sb for Cu in Cu_2_Ga_4_Te_7_, to gain further insight into the potential effect on physical properties. Through Sb replacement, the nominal compounds Cu_2−*x*_Sb_*x*_Ga_4_Te_7_ crystallize with the crystal structure of CuGaTe_2_ with the real compositions deviating from those of their nominal ones. Besides, an addition of Sb increases the effective mass (m*) of the carrier. Coupled with the enhancement in carrier concentration and phonon–electron interactions, the TE performance was improved.

## 2. Experimental

### 2.1 Sample preparation

Four elements (Cu, Ga, Te, and Sb) (Emei Semicon. Mater. Co., Ltd. Sichuan, CN), with purities of more than 99.999%, were loaded into different vacuum silica tubes according to the formula Cu_2−*x*_Sb_*x*_Ga_4_Te_7_ (*x* = 0.05, 0.1, 0.2) and then melted at 1373 K. When they were melted, the samples were rocked for 30 s every 1 h to ensure a homogeneous composition without segregation. After cooling down from 1373 K to room temperature (RT), the solidified ingots were pulverized and then ball-milled at a rotation rate of 350 rpm for 5 h in stainless steel bowls that contained benzinum. Subsequently, the dried powder was quickly sintered by using spark plasma sintering apparatus (SPS-1030) at a peak temperature of 823 K and a pressure of 55 MPa. The holding time at 823 K was controlled to be ∼2 min. The densities (*d*) of the polished bulks, which were more than 95% of the theoretical density (5.84 g cm^−3^),^24^ were measured using Archimedes’ method. Pristine Cu_2_Ga_4_Te_7_ (*x* = 0) was also prepared for comparison.

Bulk samples with sizes of 2.5 × 3 × 12 mm^3^ and 2 × 2 × 7 mm^3^ were prepared for the measurement of electrical properties and Hall coefficients respectively, and those of *ϕ* 10 × 1.5 mm^2^ for thermal diffusivity measurements.

### 2.2 Physical property measurements

The physical parameters, which involve Seebeck coefficients (*α*) and electrical conductivities (*σ*) as a function of temperature, were measured under a helium atmosphere from RT to ∼805 K in a ULVAC ZEM-3 instrument system with an uncertainty of 6.0% for each. The thermal diffusivities were measured by using TC-1200RH apparatus from RT to ∼ 805 K. Owing to the lower than RT Debye temperature of Cu_2_Ga_4_Te_7_ (222 K, ref. 28), the Dulong–Petit rule is viable to estimate the heat capacities (*C*_p_) above RT.^23^ The thermal conductivities (*κ*) were then directly calculated as the products of material densities (*d*), specific heats (*C*_p_) and thermal diffusivities (*κ*). The lattice contributions (*κ*_L_) were obtained by subtracting the electronic part (*κ*_e_) from the total *κ*, *i.e.*, *κ*_L_ = *κ* − *κ*_e_, here *κ*_e_ is expressed by the Wiedemann–Franz law, *κ*_e_ = *L*_0_*σT*, where *L*_0_ is the Lorenz number, estimated at 2.45 × 10^−8^ W Ω K^−2^ for degenerate environments of semiconductors.^29^ The three parameters (*α*, *σ*, and *κ*) were finalized by taking the average values of several samples tested by the same method.

Hall coefficients (*R*_H_) were measured by using a four-probe configuration in a system (PPMS, Model-9) with a magnetic field up to ±2 T. The Hall mobility (*μ*) and carrier concentration (*n*_H_) were calculated according to the relations *μ* = |*R*_H_|*σ* and *n*_H_ = 1/(*eR*_H_) respectively, where *e* is the electron charge.

### 2.3 Chemical compositions and structural analyses

Structural analysis of the powders was made by using a powder X-ray diffractometer (D8 Advance) operating at 50 kV and 40 mA with Cu Kα radiation (*λ* = 0.15406 nm) in the range from 10° to 110° with a step size of 0.02°, and an X’Pert Pro, PANalytical code was used to do the Rietveld refinement of the XRD patterns of the titled compounds. The lattice constants *a* and *c* were directly obtained from the refinement of the XRD patterns using Jade software.

The chemical compositions of the samples Cu_2−*x*_Sb_*x*_Ga_4_Te_7_ (*x* = 0, 0.2) were checked using an electron probe micro-analyzer (EPMA) (S-4800, Hitachi, Japan) with an accuracy of >97%.

## 3. Results and discussions

### 3.1 Composition analyses and XRD

Fig. S1† shows the EMPA mappings of four elements, Cu, Sb, Te and Ga, for the sample at *x* = 0.2. The average chemical compositions of stoichiometric Cu_2_Ga_4_Te_7_ and Cu_1.8_Ga_4_Sb_0.2_Te_7_ are shown in Table S1,† where the number of moles of Te was normalized to 7.0. Generally, the relative molar fractions shown in Table S1† are close to those of the nominal ones, and the four elements are distributed relatively uniformly in the matrix without much segregation, indicating that the titled materials were well prepared.

**Fig. 1:**
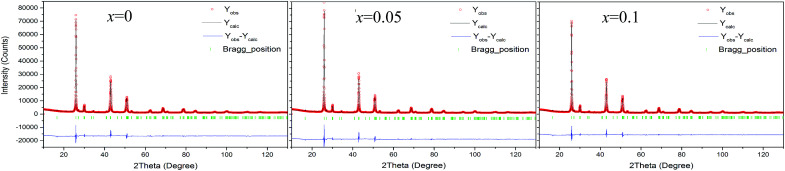
Rietveld refinements using X-ray diffraction data of the three compounds Cu_2−*x*_Sb_*x*_Ga_4_Te_7_ (*x* = 0, 0.05, and 0.1).

**Fig. 2:**
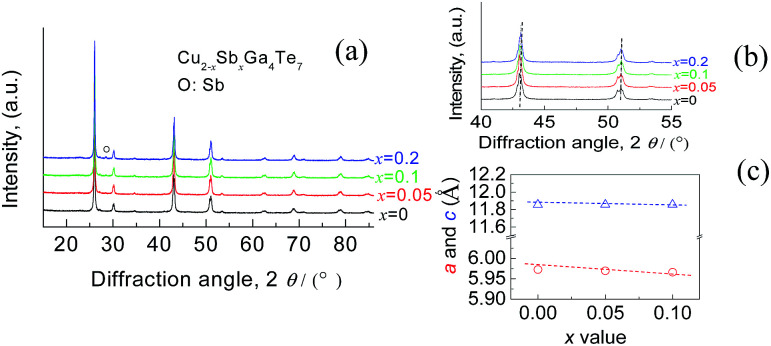
(a) XRD patterns of the powders Cu_2−*x*_Sb_*x*_Ga_4_Te_7_ (*x* = 0–0.1); (b) close-up view of XRD patterns between 40° and 55°; (c) refined lattice constants *a* and *c* as a function of the *x* value, with an analysis error of <0.4%.

The Rietveld refinement using the XRD data of three compounds Cu_2−*x*_Sb_*x*_Ga_4_Te_7_ (*x* = 0, 0.05, and 0.1) was conducted, and the results are shown in Fig. 1. Here we did not present the refined XRD data of the compound at *x* = 0.2 due to abnormal SOFs (site of occupation factors) and big *S* (goodness of fit indicator) values, likely caused by the precipitation of the visible impurity, Sb (see the XRD patterns of the powders in Fig. 2(a)). Although the precipitation of impurities does not affect the overall compositions, it is noted that the nominal compounds Cu_2−*x*_Sb_*x*_Ga_4_Te_7_ (*x* = 0, 0.05 and 0.1) actually crystallize in a crystal structure of CuGaTe_2_ (PDF, 79-2331(122), s.g.: *I*4̄2*d*), and the real compositions from refinement are Cu_0.714_GaTe_2_, Cu_0.696_Sb_0.018_GaTe_2_ and Cu_0.68_Sb_0.034_GaTe_2_ respectively. The deviation of the refined compositions from the nominal ones was highly likely, and can be assumed to be caused by the precipitation of impurity phases when some element contents exceeded their solubilities at certain temperatures. Because of the low analyzing accuracy of XRD analysis, some tiny secondary phases are hard to identify.

**Fig. 3:**
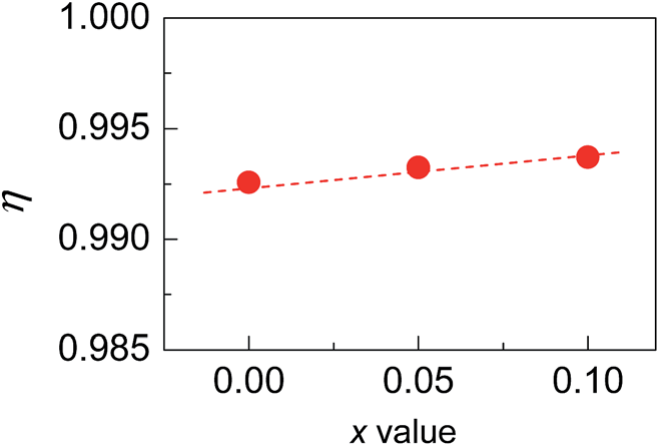
The lattice distortion parameter *η* in Cu_2−*x*_Sb_*x*_Ga_4_Te_7_ (*x* = 0–0.1) with a chalcopyrite structure as a function of Sb content (*x* value).

Shown in Fig. 2(b) is a close-up view of the XRD patterns between 40° and 55°, where the peak positions tend to shift toward large angles, indicating the shrinkage of the crystal lattice. The lattice constants *a* (5.9662–5.9724) and *c* (11.8570–11.8587) against the Sb content (*x* value), taken from the refined results shown in Table 1, are presented in Fig. 2(c). The structural variables in Table 1, such as *R*_B_ (Bragg factor), *R*_p_ (profile factor), *R*_wp_ (weighted profile factor), and *S*, are in the ranges of 7.02–7.89%, 5.22–5.24%, 6.77–7.28%, and 2.73–2.91 respectively. Although the *S* values seem a little large, they are comparable to those from the refinements on Cu_18_Ga_25_Te_50_ (ref. 21) and Cu_3_In_5_Te_9_.^30^ Besides, the Wyckoff positions, atomic coordinates and SOFs are presented in Table 2. The SOF values indicate that the element Sb totally resides in the Cu 4b site with SOFs of 0.018 (*x* = 0.05) and 0.034 (*x* = 0.1) respectively, while Ga and Te atoms reside in 4a and 8d sites (SOFs = 1). Fig. 3 represents the lattice structure distortion parameter *η*, where *η* = *c*/2*a*, against the *x* value for the ternary chalcopyrite compounds. The *η* value tends to increase and approaches 1.0 as the *x* value increases, indicating that the crystal structure distortion gets weakened as more Sb is added.

**Table 1:** Refined structure parameters of Cu_2−*x*_Sb_*x*_Ga_4_Te_7_ (*x* = 0, 0.05, and 0.1)

	*x* = 0	*x* = 0.05	*x* = 0.1
Chemical formula	(Cu_0.714_GaTe_2_)	(Cu_0.696_Sb_0.018_GaTe_2_)	(Cu_0.680_Sb_0.034_GaTe_2_)
Space group	*I*4̄2*d* (no. 122)	*I*4̄2*d* (no. 122)	*I*4̄2*d* (no. 122)
*Z*	4	4	4
*a* (Å)	5.9724 (3)	5.9697 (3)	5.9662 (3)
*b* (Å)	5.9724 (3)	5.9697 (3)	5.9662 (3)
*c* (Å)	11.8570 (1)	11.8587 (9)	11.8580 (1)
*V* (Å^3^)	423.00 (5)	422.61 (4)	422.09 (5)
*R*_B_ (%)	7.02	7.56	7.89
*R*_p_ (%)	5.22	5.64	5.40
*R*_wp_ (%)	6.77	7.28	6.89
*S*	2.91	2.73	2.83

**Table 2:** Wyckoff positions, atomic coordinates, and occupancies of Cu_2−*x*_Sb_*x*_Ga_4_Te_7_

Compositions	Atom	Site	*x*	*y*	*z*	*B*_iso_ (Å^2^)	Occupancy
*x* = 0	Cu	4b	0	0	0.5	0.5(2)	0.714(1)
	Ga	4a	0	0	0	0.9(2)	1
	Te	8d	0.265(6)	0.25	0.125	1.22(2)	1
*x* = 0.05	Cu	4b	0	0	0.5	0.9(3)	0.696(1)
	Sb	4b	0	0	0.5	0.9(3)	0.018(1)
	Ga	4a	0	0	0	0.9(2)	1
	Te	8d	0.2643(7)	0.25	0.125	1.29(3)	1
*x* = 0.1	Cu	4b	0	0	0.5	1.3(2)	0.68(1)
	Sb	4b	0	0	0.5	1.3(2)	0.034(1)
	Ga	4a	0	0	0	0.9(1)	1
	Te	8d	0.2645(8)	0.25	0.125	1.10(2)	1

### 3.2 Transport properties

**Fig. 4:**
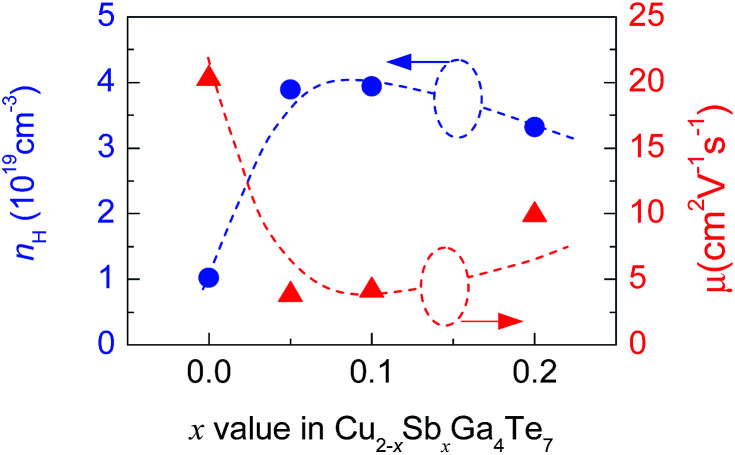
The Hall carrier concentration (*n*_H_) and mobility (*μ*) at RT of Cu_2−*x*_Sb_*x*_Ga_4_Te_7_ (*x* = 0–0.2) compounds as a function of Sb content (*x* value).

The measured Hall coefficients (*R*_H_) are positive, indicating that the materials exhibit p-type semiconducting behavior. The calculated Hall carrier concentration (*n*_H_) and mobility (*μ*) at RT are shown in Fig. 4. Upon Sb incorporation, the *n*_H_ value grows from 1.02 × 10^18^ cm^−3^ (*x* = 0) to 3.89 × 10^19^ cm^−3^ (*x* = 0.05) as Sb content increases, and then it reduces to 3.32 × 10^19^ cm^−3^(*x* = 0.2). The *μ* value reduces drastically from 20.3 cm^2^ V^−1^ s^−1^ (*x* = 0) to 3.9 cm^2^ V^−1^ s^−1^ (*x* = 0.05) followed by an increasing tendency. At *x* = 0.2, the *μ* value is 9.9 cm^2^ V^−1^ s^−1^. These results imply that the transport properties (*n*_H_ and *μ*) of carrier are very sensitive to Sb incorporation in Cu_2_Ga_4_Te_7_. However, after incorporation of a small amount of Sb (*x* = 0.05) in the Cu site, the carrier transport becomes relatively inactive, and only small changes in the *n*_H_ (*μ*) value were observed as the Sb content increases. The reason for this might be that the Sb_Cu_ defect provides two extra electrons which neutralize the p-type holes. On the other hand, the slight changes in *n*_H_ and *μ* at *x* ≥ 0.05 imply that alteration of the chemical environment plays a minor role, based on the estimation made using the valence count rule^31,32^ (the results are not shown here). In this regard, the origin of the enhancement in *n*_H_ might be due to the unpinning of the Fermi level followed by its movement into the inner side of the valence band as Sb occupies the Cu site, as is observed in Sb-substituted Cu_18_Ga_25_Te_50_.^21^

### 3.3 TE performance

**Fig. 5:**
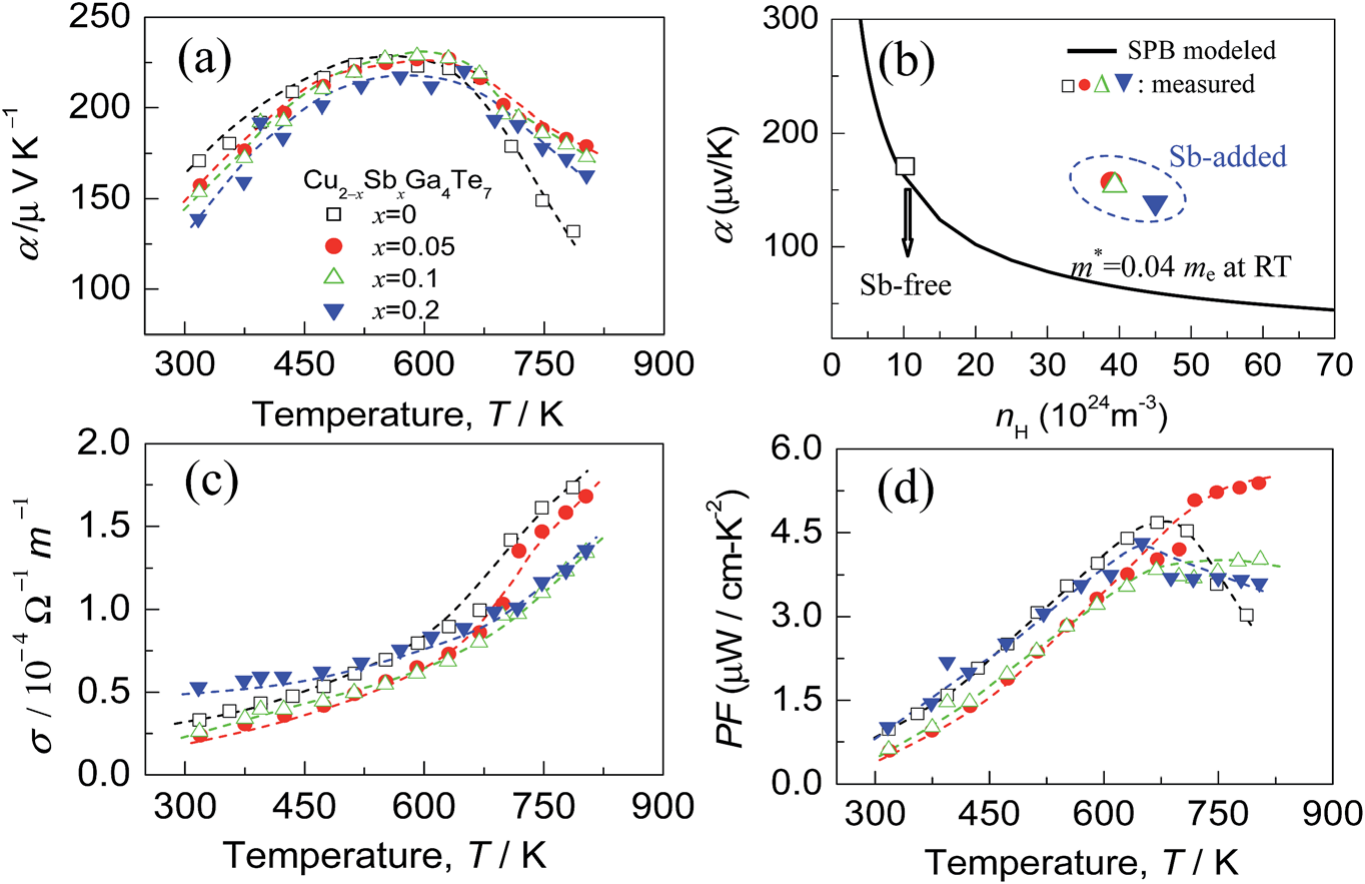
(a) Seebeck coefficients (*α*) of the compounds Cu_2−*x*_Sb_*x*_Ga_4_Te_7_ (*x* = 0.05, 0.1 and 0.2) as a function of temperature, and that for *x* = 0 is presented for comparison; (b) the experimentally determined Seebeck coefficients (*α*) at the corresponding Hall carrier concentrations, labeled by 
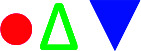
. The solid line represents the Pisarenko relation at RT; (c) electrical conductivities (*σ*) as a function of temperature for different materials (*x* values); (d) power factor PF, PF = *α*^2^*σ*, for different materials (*x* values).

The Seebeck coefficients (*α*) of the Cu_2−*x*_Sb_*x*_Ga_4_Te_7_ (*x* = 0–0.2) compounds as a function of temperature are presented in Fig. 5(a). The *α* values, which are positive, increase as the measured temperature increases, until the peak temperature (∼600 K) is reached. After that, they start to decrease with increasing temperature. Above ∼700 K, the *α* values at *x* ≥ 0.05 are much higher than those of the Sb-free sample (*x* = 0). This might be the result of the dominant increase of effective mass. In order to substantiate this assumption, the dependence of the Seebeck coefficients on the Hall carrier concentration is depicted in Fig. 5(b), assuming that the Pisarenko relation^26,33^ with the SPB model is valid in the Cu–Ga–Te systems.^34–36^ This dependence indicates that the *α* values of the Sb-incorporated samples (circled by dotted line) are much higher than those predicted by the Pisarenko relation at the corresponding carrier concentrations. The solid line depicted in Fig. 5(b) corresponds to the relationship between *α* and *n*_H_ for the Sb-free Cu_2_Ga_4_Te_7_ at RT with an effective mass of m* = 0.04*m*_e_. It is therefore determined that the effective carrier mass increases upon Sb incorporation (see the further discussion below). Besides, as Sb content (*x* value) increases, the electrical conductivity (*σ*) has a slight decrease over the whole temperature range (see Fig. 5(c)), and at ∼800 K the *σ* value decreases from 1.73 × 10^4^ Ω^−1^ m^−1^ (*x* = 0) to 1.68 Ω^−1^ m^−1^ (*x* = 0.05) and 1.34 × 10^4^ Ω^−1^ m^−1^ (*x* = 0.2). The power factors (PF), PF = *α*^2^*σ*, are presented in Fig. 5(d). It was observed that the highest PF value for the Sb-free sample is 4.67 μW cm^−1^ K^−2^ at ∼675 K, while that at *x* = 0.05 is 5.39 μW cm^−1^ K^−2^ at ∼800 K, increasing by 16%. Owing to the degradation in electrical conductivity at high temperatures as Sb content increases, it is believable that the enhancement in power factor above ∼700 K is mainly attributed to the increased *α* values.

**Fig. 6:**
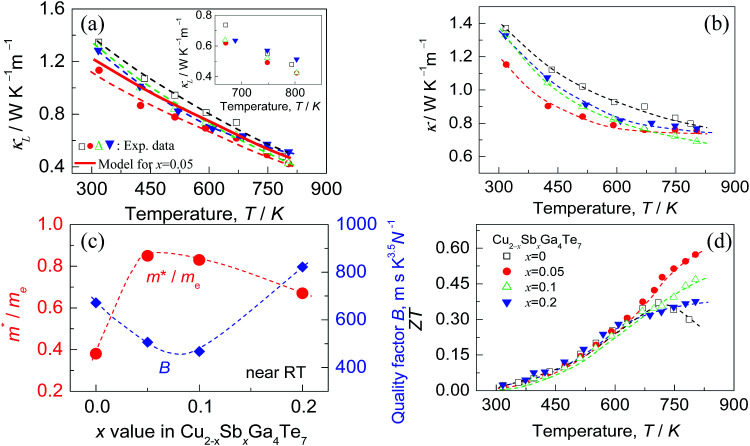
(a) Lattice thermal conductivities (*κ*_L_) as a function of temperature for different materials (*x* values). The solid red line represents the fitted results for the sample at *x* = 0.05 using the Callaway and Klemens model. The inset is a close-up view of the *κ*_L_ values at high temperatures; (b) total thermal conductivities (*κ*) as a function of temperature for different materials (*x* values); (c) the m*/*m*_e_ and quality factor *B* values as a function of Sb content (*x* value); (d) TE figure of merit (*ZT*) as a function of temperature for different materials (*x* values).

Shown in Fig. 6(a) are the lattice thermal conductivities (*κ*_L_) against temperature for Cu_2−*x*_Sb_*x*_Ga_4_Te_7_ (*x* = 0–0.2). The *κ*_L_ values reduce with temperature increasing, roughly obeying the *T*^−1^ relation. The *κ*_L_ value at *x* = 0 is higher than those at *x* = 0.05 and 0.1 over the whole temperature range, as shown in Fig. 6(a) as an inset. The total thermal conductivities (*κ*) at *x* = 0 remain high compared with those of the Sb-incorporated samples (Fig. 6(b)), partly due to high electronic contributions (*κ*_e_). In addition, the m*/*m*_e_ value increases with increasing *x* value until *x* = 0.05, and then it starts to decrease, as shown in Fig. 6(c). However, the quality factor *B* (*B* = *μ*_H_(m*/*m*_e_)^3/2^*T*^5/2^/*κ*_L_)^33^ exhibits an opposite trend to the effective mass. The *B* value decreases with increasing Sb content until *x* = ∼0.07, and then increases rapidly. Combined with the three physical parameters (*α*, *σ*, and *κ*) measured, we attained the TE figure of merit (*ZT*), as shown in Fig. 6(d). At *x* = 0.05 the highest *ZT* value is 0.58 at ∼803 K, which is about 0.21 higher than that of the pristine Cu_2_Ga_4_Te_7_ (*ZT* = 0.37).

In brief, the improvement in TE performance is attributed to two main aspects: increased Seebeck coefficient, and reduced lattice thermal conductivity.

In general, at a temperature far above the Debye temperature, all phonon modes are activated. In the present nominal compounds Cu_2−*x*_Sb_*x*_Ga_4_Te_7_ with chalcopyrite structure, the lattice thermal conductivity is governed by complex scattering mechanisms, such as, the lattice disorder scattering, Umklapp scattering, phonon–electron scattering, and the extra scattering caused by the crystal structure distortion. However, the scattering caused by the crystal structure distortion should be decreased, because the distortion parameter *η* has an increasing tendency (approaching 1.0) (see Fig. 3), based on the previous investigations.^37–39^ While the extra scattering resulted from the created Sb_Cu_ defect should be larger, since the regular arrangement of the one-seventh cation vacancies in the Cu_2_Ga_4_Te_7_ system^28^ suffers disturbance when Sb resides in the Cu site (Table 2), attributed to the differences in atomic size and electronegativity between Sb (1.53 Å, 2.05) and Cu (1.57 Å, 1.9).^40^ That is why we have observed a general reduction in *κ*_L_ as the Sb content increases. The high *κ*_L_ values at *x* = 0.2 at high temperatures might be due to the donor Sb_Cu_ defect along with the visible Sb impurity neutralizing the inherent p-type cation vacancy, thus reducing the vacancy scattering centers of phonons.^27^

In order to substantiate the general reduction in *κ*_L_ as Sb content increases, we estimated the *κ*_L_ values by means of the Callaway and Klemens model^41–43^ to outline the contributions from the Umklapp and point defect scatterings.^34,44^ When estimating *κ*_L_ using this model, the ratio of the modeled lattice thermal conductivity of the crystal with Sb substitution for Cu, *κ*_L_^m^, to the lattice thermal conductivity of the pure crystal, *κ*_L_^p^, is given below,1
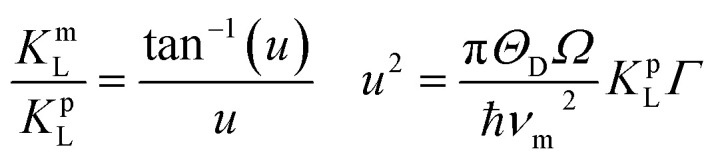
where *u* and *Γ* are the disorder scaling parameter and the disorder scattering parameter respectively. Here we use the factor *Γ* below to predict the *κ*_L_ values for the Cu–Ga–Te based chalcogenides,^34^2
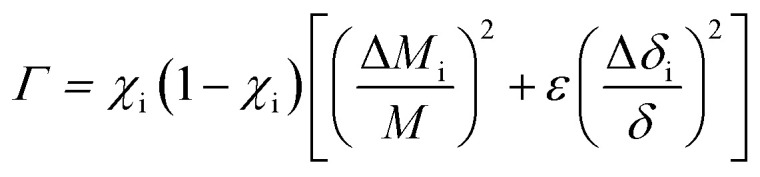
where *χ*_i_, Δ*M*_i_/*M* and Δ*δ*_i_/*δ* are the molar fraction of Sb, relative change of atomic mass due to the replacement of Cu by Sb, and the local change in lattice parameter. *ε* = 2(*W* + 6.4*γ*)^2^ is determined by using the Grüneisen parameter *γ* = 1.46 and *W* = 3.^34^ The other related parameters are presented in Table 3.

**Table 3:** Parameters used for estimating the lattice thermal conductivity (*κ*_L_) at *x* = 0.05 using the Callaway and Klemens model

Symbol	Representation
*Θ*_D_	Debye temperature, 222 K (ref. 28)
*ν*_m_	Average sound velocity, 2227 m s^−1^ (ref. 25 and 45)
*ℏ*	Planck constant
*Ω*	Volume per atom

The fitting results for the sample at *x* = 0.05 using the above model are shown in Fig. 6(a) as a red solid curve. Roughly, the estimated *κ*_L_ values follow the same decreasing trend as the experimental data over the whole temperature range, which confirms the importance of the Umklapp and point defect scattering mechanisms. However, it is worth noting that the estimated *κ*_L_ values are a little higher than the measured ones, suggesting that the Umklapp and point defect scattering are not enough to account for the reduction in *κ*_L_, although the fitting may introduce some error using 222 K as the Debye temperature.^28^ It is therefore believed that there exists another phonon scattering mechanism, that is, the phonon–electron scattering, due to an enhanced carrier concentration upon Sb incorporation. This scattering plays a major role in further reducing *κ*_L_. Here it should be pointed out that we did not estimate the *κ*_L_ values of the samples at *x* = 0.1 and 0.2, because the Debye temperature (222 K) of these materials might change significantly. Therefore, it is not suitable for further estimations to be made.

## 4. Conclusions

The Cu_2−*x*_Ga_4_Sb_*x*_Te_7_ ternary compounds with Sb substituted for Cu were prepared and their TE properties examined. Rietveld refinement reveals that these compounds (*x* = 0, 0.05 and 0.1) actually crystallize in a crystal structure of CuGaTe_2_, and the real compositions are Cu_0.714_GaTe_2_, Cu_0.696_Sb_0.018_GaTe_2_ and Cu_0.68_Sb_0.034_GaTe_2_ respectively. Besides, Sb is incorporated into the Cu site, which is responsible for the enhancement in Hall carrier concentration (*n*_H_) and the decrease in mobility (*μ*). In addition, the Seebeck coefficient increases above ∼700 K, due to an increase of the effective carrier mass. The reduction in lattice thermal conductivity (*κ*_L_) is closely related to the increase in point defect and phonon–electron scattering as the Sb content increases. As a consequence, the highest *ZT* value of 0.58 is reached at ∼803 K for the Cu_1.95_Ga_4_Sb_0.05_Te_7_ sample, which is about 0.21 higher than that of the pristine Cu_2_Ga_4_Te_7_ (*ZT* = 0.37).

## Conflicts of interest

There are no conflicts to declare.

## Supplementary Material

RA-008-C8RA03704C-s001
