# Supplementary material for: Increased effective mass and carrier concentration responsible for the improved thermoelectric performance of the nominal compound Cu2Ga4Te7 with Sb substitution for Cu
Source: RSC Adv. 2018 Jun 14;8(38):21637–43. doi: 10.1039/c8ra03704c (PMC9080934; doi:10.1039/c8ra03704c)
Supplement: RA-008-C8RA03704C-s001 [file RA-008-C8RA03704C-s001.pdf]

Electronic Supplementary Material (ESI) for RSC Advances.
This journal is © The Royal Society of Chemistry 2018

Supporting information

Increased effective mass and carrier concentration responsible for the improved thermoelectric performance of $Cu_2Ga_4Te_7$ with Sb substitution for Cu

Jiaolin Cui,[a] * Gemei Cai, [b] Wei Ren[a]

Jiaolin Cui, Wei Ren: School of Materials & Chemical Engineering, Ningbo University of Technology, Ningbo 315016, China

Gemei Cai: School of Materials Science and Engineering, Central South University, Changsha 410083, China

Corresponding author's contact information:

Jiaolin Cui:

School of Materials and Chemical Engineering, Ningbo University of Technology, Ningbo 315211, China

E-mail: cuijiaolin@163.com

Tel: 86-574-87080504

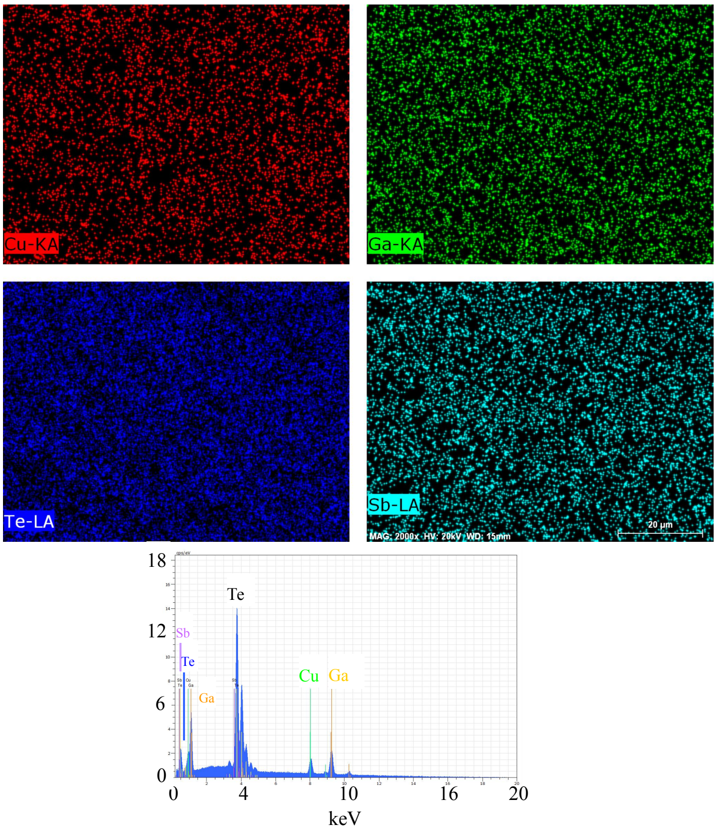

Fig. S1 EPMA mappings of four elements on polished $Cu_{1.8}Sb_{0.2}Ga_4Te_7$ surface (a) Cu, (b) Ga, (c) Te and (d) Sb, (e) an EDAX pattern.

Table S1 Average chemical compositions (relative molars) identified for $Cu_2Ga_4Te_7$ and $Cu_{1.8}Sb_{0.2}Ga_4Te_7$ (taken from different mappings)

Compounds	Cu	Ga	Sb	Te
$Cu_2Ga_4Te_7$	1.97	4.03	---	7.0
$Cu_{1.8}Sb_{0.2}Ga_4Te_7$	1.78	4.05	0.15	7.0
